# Intraoperative radiotherapy boost as part of breast-conservation therapy for breast cancer: a single-institution retrospective analysis

**DOI:** 10.1007/s00066-021-01785-2

**Published:** 2021-04-30

**Authors:** Raluca Stoian, Thalia Erbes, Constantinos Zamboglou, Jutta Scholber, Mark Gainey, Ilias Sachpazidis, Erik Haehl, Simon K. B. Spohn, Vivek Verma, David Krug, Alexander Rühle, Ingolf Juhasz-Böss, Anca-Ligia Grosu, Nils H. Nicolay, Tanja Sprave

**Affiliations:** 1 Department of Radiation Oncology, University Hospital of Freiburg, Robert-Koch-Straße 3, 79106 Freiburg, Germany; 2 German Cancer Consortium (DKTK) Partner Site Freiburg, German Cancer Research Center (dkfz), Neuenheimer Feld 280, 69120 Heidelberg, Germany; 3 Department of Obstetrics and Gynecology, Medical Center, University of Freiburg, Freiburg, Germany; 4 Faculty of Medicine, University of Freiburg, Freiburg, Germany; 5 Department of Radiation Oncology, Allegheny General Hospital, Pittsburgh, PA USA; 6 Department of Radiation Oncology, University Hospital Schleswig-Holstein, Arnold-Heller-Str. 3, 24105 Kiel, Germany; 7 Department of Molecular and Radiation Oncology, German Cancer Research Center (dkfz), Neuenheimer Feld 280, 69120 Heidelberg, Germany

**Keywords:** Early breast cancer, Breast-conservation therapy, Adjuvant radiation therapy, Boost, Intraoperative radiotherapy

## Abstract

**Background:**

There are currently no data from randomized controlled trials on the use of intraoperative radiotherapy (IORT) as a tumor bed boost as part of a breast-conservation approach for breast cancer. This study retrospectively reviewed the safety and efficacy of IORT as a boost treatment at a tertiary cancer center.

**Methods:**

From 2015 to 2019, patients underwent breast-conserving surgery with axillary lymph node staging and a single dose of 20 Gy IORT with 50-kV photons, followed by whole-breast irradiation (WBI) and adjuvant systemic therapy (if applicable). Patients were followed for assessment of acute and late toxicities (using the Common Terminology Criteria for Adverse Events version 5.0) at 3–6-month intervals. Outcomes included ipsilateral (IBTR) and contralateral breast progression-free survival (CBE), distant metastasis-free survival (DMFS), and overall survival (OS).

**Results:**

Median follow-up for the 214 patients was 28 (range 2–59) months. Most patients had T1 disease (*n* = 124) and were clinically node negative. Only few patients had high-grade and/or triple-negative disease. The vast majority of patients underwent sentinel node biopsy, and 32 (15%) required re-resection for initially positive margins. Finally, all tumor bed margins were clear. Nine (4.2%) and 48 (22.4%) patients underwent neoadjuvant and adjuvant chemotherapy, respectively. WBI was predominantly performed as conventionally fractionated WBI (*n* = 187, 87.4%), and the median time from BCS to WBI was 54.5 days. IORT was delivered with a single dose of 20 Gy. The median WBI dose was 50 Gy (range 29.4–50.4 Gy). No patients experienced grade 4 events; acute grade 3 toxicities were limited to 17 (8%) cases of radiation dermatitis. Postoperative toxicities were mild. After WBI only one case of late grade ≥ 2 events was reported. There were two recurrences in the tumor bed and one contralateral breast event.

**Conclusion:**

This investigation provides additional preliminary data supporting the using of IORT in the boost setting and corroborates the existing literature. These encouraging results should be prospectively validated by the eventual publication of randomized studies such as TARGIT‑B.

## Introduction

Following breast-conserving surgery (BCS), radiotherapy (RT) reduces local recurrence and breast cancer mortality [1]. There is an additional reduction in local recurrence with the delivery of a focal RT boost to the tumor bed following whole-breast irradiation (WBI) [2]. External-beam RT is the most commonly used technique for adjuvant treatment of breast cancer; however, other modalities have been increasingly explored.

Intraoperative RT (IORT) is one such modality that has been used as a substitute for both WBI as well as in the boost setting [3, 4]. Randomized data for the former setting include the TARGIT‑A and ELIOT trials [5, 6], and the accruing TARGIT‑B (NCT01792726) and recently published HIOB (NCT01343459) [7] trials. IORT is delivered by means of electrons or 50-kV X‑ray therapy as a single fraction during the course of BCS.

IORT has been primarily analyzed for patients with low-risk features. The sole use of IORT as a substitute for WBI was critically questioned. The current data demonstrate a tendency for higher local recurrence rates; therefore, the authors recommend critical patient selection for the use of IORT alone when omitting WBI [8].

IORT has several theoretical advantages, in particular “same-day approach” settings in addition to increased patient convenience [9]. IORT allows for increased skin sparing and avoids potential tumor repopulation between the completion of surgery and initiation of WBI. It also allows for a more accurate assessment of the dimensions of the operative tumor bed and, hence, potentially reduces irradiated volumes. In comparison to the scar boost using electrons, the risk of a potential target miss due to problems with identification of the tumor bed can be avoided. Notably, current guidelines allow a safe and reproducible boost definition even after the oncoplastic surgical procedures [10].

In the absence of published data from randomized trials of IORT as a boost, reporting institutional experiences is necessary. The goal of this single-institutional retrospective study was to describe outcomes and toxicities of IORT boost using 50-kV X‑rays for early breast cancer.

## Materials and methods

### Patients and treatment

From 2015 to 2019, breast cancer patients treated with a single dose of 20 Gy IORT as a tumor bed boost were included in this analysis. Institutional criteria for patient selection for IORT boost included patients with BCS, premenopausal status, postmenopausal in addition to the following risk factors: tumor size ≥ 2 cm, extensive intraductal component, G3, HER2-positive, and triple-negative breast cancer (TNBC).

BCS with sentinel lymph node excision or axillary nodal dissection was performed according to institutional protocols. Neoadjuvant or adjuvant chemotherapy as well as endocrine therapy was administered based on the currently valid guidelines and individual recommendations of the interdisciplinary oncological board.

**Fig. 1:**
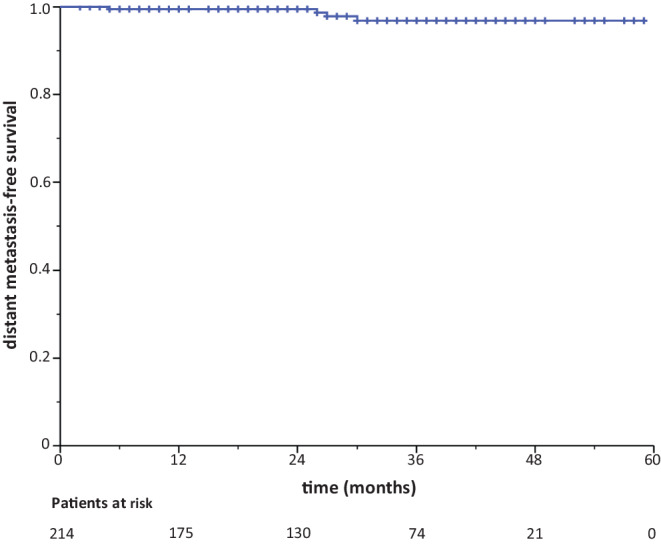
Kaplan–Meier curve for distant metastasis-free survival

IORT was performed with the following methodology: following wide tumor excision, a single IORT dose was prescribed to the applicator surface (range 20–50 mm) and skin-sparingly applied using 50-kV X‑rays with the INTRABEAM miniature X‑ray generator (Carl Zeiss Surgical, Oberkochen, Germany) (Fig. 1). IORT boost with a single dose of 20 Gy at the surface of the IORT applicator using 50-kV photons is attenuated down to 5 Gy at 1 cm distance from the edge of the excision cavity.

Special care was taken to minimize skin exposure during IORT. Subsequently, after completion of wound healing, WBI was delivered according to standardized institutional protocols, using conventional fractionation (50–50.4 Gy in 25–28 fractions) or hypofractionation (40.05 Gy in 15 fractions). Depending on the time period—beginning in 2017 was increasingly used for adjuvant WBI). CT-based (Brilliance, CT Big Bore, Philips, Cleveland, OH) three-dimensional treatment planning (Oncentra MasterPlan, Nucletron, Veenendaal, the Netherlands and/or Eclipse™ planning systems, Varian Medical Systems) was performed using standard tangential treatment portals (6 or 18 MV; Synergy; Elekta, Crawley, United Kingdom). From 2017, patients with left-sided breast cancer received WBI in deep-inspirational breath-hold (DIBH) technique with Surface Image Guided RT (C-RAD, Catalyst, C‑RAD AB, Uppsala, Sweden). In some cases with anatomical variations, an intensity-modulated RT (IMRT) or volumetric modulated arc therapy (VMAT) technique was used to reduce the lung and heart dose.

Systemic therapy was administered according to national and international guidelines (the Gynecologic Oncology Working Group recommendations, S3 guideline, St. Gallen consensus). Following WBI, patients with estrogen receptor (ER)-positive disease received 5–10 years of adjuvant endocrine therapy. Breast ultrasound was performed every 6 months for the first 3 years. Mammograms were obtained 6 months after WBI, and yearly after the first mammography. Suspected recurrences were biopsy confirmed.

All patients were followed-up every 3–6 months for the first 2 years in the radiation oncology department, followed by annual visits thereafter. Acute toxicity assessments were conducted at each visit according to the Common Terminology Criteria for Adverse Events (CTCAE) version 5.0. Late toxicity was assessed by the treating radiation oncologists and gynecologist based on modified Late Effects in Normal Tissues criteria (Subjective, Objective, Management, and Analytic, LENT-SOMA). For this purpose, the planning CT scans and ultrasound examinations were evaluated. According to this, seroma and hematoma were classified as grade 1 (asymptomatic), 2 (simple aspiration needed), and 3 (surgical intervention).

### Statistical analysis

Outcomes included ipsilateral breast progression-free survival (IBTR), contralateral breast progression-free survival (CBE), distant metastasis-free survival (DMFS), and overall survival (OS). All were defined from the date of IORT to the pertinent event. Survival times were calculated using the Kaplan–Meier method. Data are reported as a mean, median (range), and frequencies. Binary correlation analysis using Spearman rank correlation to examine the impact of various determinants (in particular applicator size, systemic therapy, or fractionation regimen) on acute and late toxicity was used. *P*-values < 0.05 were considered statistically significant. Statistics were performed with SPSS version 25 (IBM, Armonk, NY, USA).

## Results

Altogether, 214 patients were analyzed for this investigation. Table 1 displays clinical characteristics of this population. Most patients had T1 disease (*n* = 124) and were clinically node negative. Only few patients had high-grade and/or triple-negative disease.

**Table 1:** Patient and tumor characteristics

Treatment characteristics	
**Total *n*** **=** **214 patients**	***n*** **(%)**
BCS	214 (100)
SLND	208 (97.2)
ALND	7 (3.3)
**Resection status**	
R0	185 (86.4)
R1	23 (10.7)
Re-resection needed due to R+ status	32 (15.0)
Neoadjuvant chemotherapy	9 (4.2)
Adjuvant chemotherapy	48 (22.4)
**Endocrine therapy**	208 (97.2)
Simultaneous	9 (4.3)
Adjuvant	53 (25.5)
Upfront	146 (70.2)
**IORT dose**	
20 Gy	214 (100)
Applicator surface median (mm)	35 (20–50)
**WBI**	
Normofractionated (25–28 ×)	187 (87.4)
Hypofractionated (15 ×)	25 (11.7)
Not applied	2 (0.9)
3DRT	200 (94.3)
IMRT/VMAT	12 (5.7)
DIBH	33 (15.6)
Median time (days) from IORT to WBI (range)	54.5 (21–325)
***Regional nodal irradiation***	
*Normo-fractionated (28* *×)*	*11 (5.1)*

Patient and tumor characteristics of patients treated by IORT and WBI in our institution between 2009 and 2019 (*n* = 214). Staging of breast cancer was based on the 7th Edition of the UICC TNM classification

*ALND* axillary lymph node dissection, *BCS* breast-conserving surgery,* DIBH* deep-inspiration breath-hold technique*, 3DRT* 3D-conformal radiotherapy, *Gy* gray, *IMRT* intensity modulated radiotherapy, *IORT* intraoperative radiotherapy, *LN* lymph node, *ME* mastectomy, *SLND* sentinel lymph node dissection, *VMAT* volumetric modulated arc therapy, *WBI* whole breast irradiation

Table 2 shows treatment-related parameters. The vast majority of patients underwent sentinel node biopsy and 32 (15%) required re-resection for initially positive margins. Re-resection for R+ was performed as part of the first surgery in 13 patients. The remaining 19 patients underwent a second procedure with re-resection. IORT was performed as part of the first surgery in each case.

**Table 2:** Treatment characteristics

Treatment characteristics	
**Total *n*** **=** **214 patients**	***n*** **(%)**
BCS	214 (100)
SLND	208 (97.2)
ALND	7 (3.3)
**Resection status**	
R0	185 (86.4)
R1	23 (10.7)
Re-resection needed due to R+ status	32 (15.0)
Neoadjuvant chemotherapy	9 (4.2)
Adjuvant chemotherapy	48 (22.4)
**Endocrine therapy**	208 (97.2)
Simultaneous	9 (4.3)
Adjuvant	53 (25.5)
Upfront	146 (70.2)
**IORT dose**	
20 Gy	214 (100)
Applicator surface median (mm)	35 (20–50)
**WBI**	
Normofractionated (25–28 ×)	187 (87.4)
Hypofractionated (15 ×)	25 (11.7)
Not applied	2 (0.9)
3DRT	200 (94.3)
IMRT/VMAT	12 (5.7)
DIBH	33 (15.6)
Median time (days) from IORT to WBI (range)	54.5 (21–325)

Treatment details for radiotherapy using IORT and WBI of breast cancer patients (*n* = 214).

*ALND* axillary lymph node dissection, *BCS* breast-conserving surgery,* DIBH* deep-inspiration breath-hold technique*, 3DRT* 3D-conformal radiotherapy, *Gy* gray, *IMRT* intensity modulated radiotherapy, *IORT* intraoperative radiotherapy, *LN* lymph node, *ME* mastectomy, *SLND* sentinel lymph node dissection, *VMAT* volumetric modulated arc therapy, *WBI* whole breast irradiation

After re-resection, all tumor bed margins were clear. Nine (4.2%) and 48 (22.4%) patients underwent neoadjuvant and adjuvant chemotherapy, respectively. The vast majority (*n* = 187, 87.4%) received conventionally fractionated WBI, and the median time from BCS to WBI was 54.5 (range 21-325) days. IORT was successfully delivered in all cases, to a median dose of 20 Gy using applicator surface median 35 mm (range 20–50 mm). WBI was applied using standard tangential treatment portals in most cases (94.2%). Only 12 patients (5.7%) received WBI using IMRT/VMAT. The median WBI dose was 50 Gy (range 29.4–50.4 Gy); 2 (0.9%) did not receive WBI. These patients renounced WBI explicitly due to own request or advanced age, while guaranteeing a close clinical follow-up and adjuvant systemic therapy. All patients with pN2a (*n* = 3) and 8 patients with pN1a (since 2017) received normofractionated RT to the regional lymph node levels with 50.4 Gy in 28 fractions.

Table 3 demonstrates the toxicity profile of the study population. No patients experienced grade 4 events, and grade 3 toxicities after WBI were limited to 17 (8%) cases of radiation dermatitis. Postoperative toxicities occurring before WBI were mild, no case of grade 2 seroma/hematoma occurred. Late toxicities were also mild, with just one case of a late grade ≥ 2 event.

**Table 3:** Toxicity

	Toxicity grade *n* (%)			
*Total* *=* *214 patients*	*0*	*1*	*2*	*3*
*Toxicity: post IORT*				
Dermatitis	211 (98.6)	3 (1.4)	–	–
Seroma/hematoma breast	192 (89.7)	22 (10.3)	–	–
Seroma/hematoma axilla	212 (99.1)	2 (0.9)	–	–
Wound infection	210 (98.1)	4 (1.9)	–	–
Wound dehiscence	210 (98.1)	4 (1.9)	–	–
*Total* *=* *212 patients*	–	–	–	–
*Acute toxicity post WBI*				
Dermatitis	13 (6.1)	100 (46.7)	82 (38.7)	17 (8.0)
Seroma/hematoma breast	182 (85.8)	29 (13.7)	1 (0.5)	–
Seroma/hematoma axilla	211 (99.5)	1 (0.5)	–	–
Wound infection	208 (98.1)	1 (1.9)	–	–
Wound dehiscence	209 (98.6)	3 (1.9)	–	–
Fatigue	161 (75.9)	50 (23.6)	1 (0.5)	–
Pain	168 (79.2)	44 (20.8)	–	–
Lymphodema	192 (90.6)	20 (9.4)	–	–
*Late toxicity post WBI*				
Dermatitis	201 (94.8)	11 (5.2)	–	–
Seroma/hematoma breast	188 (88.7)	24 (11.3)	–	–
Seroma/hematoma axilla	211 (99.5)	1 (0.5)	–	–
Wound infection	211 (99.5)	1 (0.5)	–	–
Fatigue	187 (88.2)	24 (11.3)	1 (0.5)	–
Pain	185 (87.3)	27 (12.7)	–	–
Lymphoedema	184 (86.8)	28 (13.2)	–	–

Acute and chronic radiotherapy-related toxicities after IORT and WBI according to the Common Terminology Criteria for Adverse Events (CTCAE v5.0)

*IORT* intraoperative radiotherapy, *WBI* whole breast irradiation

**Fig. 2:**
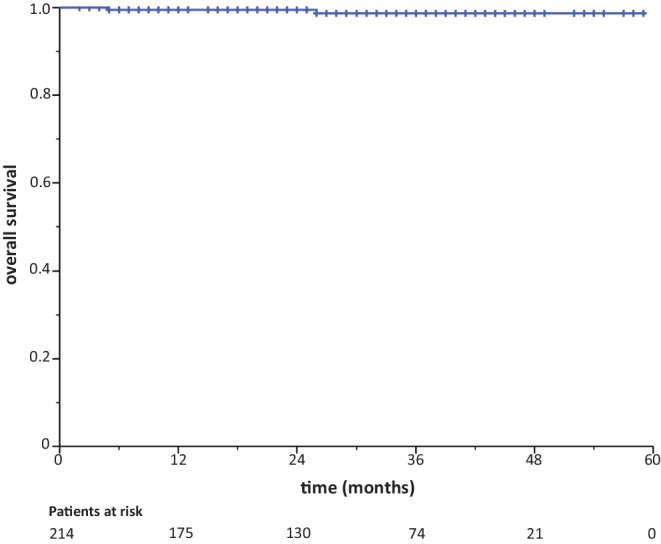
Kaplan–Meier curve for overall survival

With 28 months (range 2–59 months) median follow-up, two recurrences in the tumor bed and one contralateral breast event have occurred. The 3‑year ipsilateral breast recurrence rate (IBRT) and contralateral breast recurrence (CBE) rate were 1.8% (95% CI 99.5–92.7%) and 0.6% (95% CI 99.9–95.9%), respectively. The 3‑year DMFS and OS rates were 96.8% (95% CI 98.8–91.6%) and 98.7% (95% CI 99.7–94.7%), respectively (Figs. 1 and 2).

Lastly, binary correlation analysis did not reveal statistically significant associations between any particular variables (in particular applicator size, systemic therapy, neoadjuvant chemotherapy) and the risk of acute and chronic toxicity. Only hypofractionated WBI (Spearman rank correlation *r*_*s*_ = −0.197, *p* = 0.004, Cohen’s effect size: weak to moderate) was significantly correlated with increased occurrence of acute dermatitis.

## Discussion

This single-institutional retrospective investigation demonstrates preliminary results on IORT boost with 50-kV photons regarding the feasibility, mild toxicities, and association with appropriate early oncological outcomes for breast cancer.

There are few reports on the use of IORT as a tumor bed boost. Wenz et al. reported a clear correlation between breast volume, applicator size, and degree of fibrosis with comparable techniques [11]. The long-term analysis from the same institution after 78 months in 400 patients demonstrated on the one hand low in-breast and axillary node recurrence rates and on the other hand, few high-grade adverse events, mostly fibrosis and pain. The majority of late side effects occurred during the first 3 years after IORT and WBI [12]. In light of this, given the short follow-up time in our collective with median 28 months, chronic toxicity may be underrepresented. Furthermore Wenz et al. reported the development of higher-grade fibrosis grade II–III after a median of 36 months as an effect of a possibly shorter interval (< 30 days) between IORT and WBI [13]. These findings and recommendations to keep an interval between IORT and WBI of about 5–6 weeks were integrated into our clinical routine early on. In our cohort only 4.2% (*n* = 9) of patients had an interval of < 30 days and 12.7% (*n* = 27) of patients had a cumulative interval of < 35 days with a median interval of 54 days between IORT and WBI.

The largest experience is available for the IORT using electrons (IOERT) prescribed such a boost [14]. IOERT also offers satisfactory oncological outcomes in groups with a high risk for local relapse, like TNBC [15]. In a retrospective analysis comparing IOERT as an anticipated boost to patients treated with conventional percutaneous boost, improved local and locoregional control could also be shown in locally advanced stages (initial clinical stages II and III) after neoadjuvant chemotherapy [16].

When considering local antitumor efficacy, it is important to consider that the IORT boost with a single dose of 20 Gy at the surface of the IORT applicator using 50-kV photons is attenuated to 5 Gy at 1 cm from the edge of the excision cavity. Analyses of wound fluid from patients treated with IORT suggest that IORT has a positive effect on the tumor microenvironment. Noteworthily, a high single IORT dose elicits impact beyond the direct killing of residual tumor cells, because of influencing the microenvironment in the wound fluid through the interruption of the proliferative cytokine cascade and downregulation of the local expression of epidermal growth factor [17, 18]. Taking into account the limited spatial range and significant dose attenuation to 5 Gy at 1 cm depth from the edge of the excision cavity, it is questionable what effect level and efficacy would be expected.

The comprehensive adoption of a reconstructive approach during breast-conserving surgery is mostly termed oncoplastic surgery. This concept has been introduced to allow wide excision for BCS and simultaneously minimize cosmetic impairment [19]. Notwithstanding clip marking of the tumor bed, postoperative delineation of the tumor bed after oncoplastic surgery can be challenging and represents a risk for geographic miss [20, 21]. IO(E)RT is commonly performed prior to the reconstruction and/or remodeling procedure and enables precise and accurate dose delivery to the tumor bed.

On the basis of a low α/β ratio for breast cancer in the range of 3.5 to 4 [22–24], hypofractionation has been studied by a number of prospective phase III trials [25–31]. In our analysis, only 11.7% (*n* = 25) of patients received hypofractionated WBI. The use of IOERT boost (11.1 Gy) and hypofractionated WBI (40.5 Gy in 15 fractions) was studied within the non-randomized HIOB study [7]. The HIOB trial demonstrated an excellent acute and late toxicity profile without cosmetic impairment after 3 years of follow-up [7]. Grade II–III fibrosis in the tumor bed occurred in 6% (range 3–8.9) of patients [7]. In comparison, in our cohort, hypofractionated WBI was significantly correlated only with acute dermatitis. Another non-randomized phase II study evaluated hypofractionated WBI of 36.63 Gy in 11 fractions, followed by a sequential percutaneous boost of 13.32 Gy in 4 fractions [32]. The estimated 5‑year locoregional and distant control rates were 98% in each case. Secondary endpoints were acute and late toxicity, which were assessed with grade II–III in 30%/10% and 1%/3%, respectively [32]. Another approach to shorten overall treatment time is to use a simultaneous integrated boost (SIB). Data from three randomized controlled trials comparing normofractionated WBI with a SIB to normofractionated WBI with sequential boost have been published recently [33–35]. Oncological outcomes were comparable, and two of the three trials showed reduced acute toxicity with the SIB approach [33, 35].

Our findings are similar to those of existing data using IORT as a boost, demonstrating a 5-year IBTR rate of ≤ 5%, along with a similar rate of acute grade ≥ 3 and late grade ≥ 2 toxicities [36–43]. The rate of postoperative complications was also low in this study and numerically comparable to existing data, corroborating that IORT is safe from this standpoint as well. Our data compare favorably in terms of chronic toxicity with no case of fibrosis grade ≥ II (Table 3).

Despite these encouraging results, limitations of this investigation should be acknowledged. This first relates to the single-institutional and retrospective nature of this study, including careful patient selection for IORT, thus limiting applicability to other patient populations. Additionally, newer data have now provided more detailed information about the limited benefit/risk ratio for applying boost treatments to low-risk patients. Additionally, the short follow-up of this study limits the light shed onto toxicity events and recurrences/survival that occur in the long term. To this extent, obtaining longer-term results from this and other cohorts is essential. An important potential limitation of single-shot IORT is the ability to sufficiently cover adequate target volumes with adequate doses.

In summary, this investigation provides preliminary corroborative data for the use of IORT in the boost setting. Along with a substantially low rate of postoperative complications, there were altogether few higher-grade toxicities along with appropriate outcomes for early breast cancer. These results should be corroborated by the eventual publication of randomized studies such as TARGIT‑B.

## Abbreviations

ALND: Axillary lymph node dissection
BCS: Breast-conservating surgery
CBE: Contralateral breast progression-free survival
CI: Confidence interval
DIBH: Deep-inspiration breath-hold
DMFS: Distant metastasis-free survival
ER: Estrogen receptor
HER2: Human epidermal growth factor receptor 2
HT: Hormonal therapy
IBTR: Ipsilateral breast progression-free survival
IORT: Intraoperative radiotherapy
IOERT: Intraoperative radiotherapy using electrons
OS: Overall survival
RT: Radiotherapy
SIB: Simultaneous integrated boost
SLND: Sentinel lymph node dissection
TNBC: Triple-negative breast cancer
VMAT: Volumetric modulated arc therapy
WBI: Whole-breast irradiation
